# Unraveling the Diversity of Haemosporidians in Brazilian Non-Passerine Birds: Insights from Midwestern Brazil

**DOI:** 10.3390/pathogens14121286

**Published:** 2025-12-13

**Authors:** Morgana Maira Hennig, Luiz Gustavo Magalhães Alves, Marcela Natacha Aparecida Rocha, Victória Luiza de Barros Silva, Brenda Madruga Rosa, Rosa Helena dos Santos Ferraz, Sandra Helena Ramiro Corrêa, Érika Martins Braga, Richard de Campos Pacheco

**Affiliations:** 1 Programa de Pós-Graduação em Ciências Veterinárias (PPGVET), Faculdade de Medicina Veterinária (FAVET), Universidade Federal de Mato Grosso (UFMT), Av. Fernando Corrêa da Costa, 2367, Boa Esperança, Cuiabá 78060900, MT, Brazil; hennigmorgana@gmail.com (M.M.H.); marcelanatachavet@gmail.com (M.N.A.R.); vlbarros18@gmail.com (V.L.d.B.S.); rhsferraz@gmail.com (R.H.d.S.F.); 2 Departamento de Parasitologia, Instituto de Ciências Biológicas, Universidade Federal de Minas Gerais (UFMG), Av. Antônio Carlos, 6627, Pampulha, Belo Horizonte 31270901, MG, Brazil; luizgm01@gmail.com (L.G.M.A.); embraga@icb.ufmg.br (É.M.B.); 3 Setor de Animais Selvagens (SAS), Hospital Veterinário, Faculdade de Medicina Veterinária (FAVET), Universidade Federal de Mato Grosso (HOVET/UFMT), Av. Fernando Corrêa da Costa, 2367, Boa Esperança, Cuiabá 78060900, MT, Brazil; breenda.rosa@hotmail.com (B.M.R.); correasandrahelena@gmail.com (S.H.R.C.)

**Keywords:** avian malaria, captive birds, free-living birds, hemoparasites, *cytb* gene

## Abstract

Avian haemosporidians have been widely studied because they provide important insights into parasite distribution and diversity. However, most available data come from passerines, resulting in gaps regarding other bird groups, primarily due to the difficulty of sampling non-passerines in natural environments. Thus, we aimed to detect infections caused by *Plasmodium* spp. and *Haemoproteus* spp. through molecular and morphological analyses of blood samples from non-passerine birds in Midwestern Brazil. We evaluated 344 individuals from 60 species across 16 non-passerine orders. Among them, 18.89% (*n* = 65) were infected with haemosporidians. Molecular analyses identified four *Plasmodium* species: *P. nucleophilum*, which was detected in a broad range of host species; *P. juxtanucleare*, detected in *Gallus gallus*; *P. paranucleophilum*, found to infect *Rupornis magnirostris*; and *P. elongatum* in *Mustelirallus albicollis*. Additionally, an undescribed *Plasmodium* lineage was detected in *Nycticorax nycticorax*. We also identified four new *Haemoproteus* lineages infecting *Patagioenas picazuro*, *Asio clamator*, *Athene cunicularia*, and *Tyto furcata*. Additionally, the haplotype previously described in *Mycteria americana* was detected once more in this host. By revealing new lineages and expanding knowledge of parasite biodiversity, this study underscores the importance of non-passerine hosts and the need for further research on their evolutionary and host–parasite relationships.

## 1. Introduction

Avian hemosporidians are heteroxenous protozoa transmitted to birds by hematophagous dipterans during blood feeding. They are classified into four genera: *Plasmodium*, *Haemoproteus*, *Leucocytozoon*, and *Fallisia*. These parasites occur in nearly all zoogeographic regions, except Antarctica [1]. Among them, the genera *Plasmodium* and *Haemoproteus* are particularly significant because of their worldwide distribution and high prevalence in free-living birds [1,2]. The study of interactions between these pathogens and their avian hosts is frequently used as a model for investigating parasite–host dynamics [3].

Approximately 170 morphospecies of *Haemoproteus* and 55 species of *Plasmodium* have been described. However, genetic data are available for only 74 *Haemoproteus* and 24 *Plasmodium* species [4,5]. Considering that more than 4600 lineages of avian haemosporidians have been detected, a substantial gap remains between morphological and molecular information [6]. The Neotropical region is regarded as a hotspot for avian and hemosporidian diversity, with numerous species yet to be discovered [7]. Brazil harbors one of the world’s greatest avian diversities, with more than 1900 species recorded [8]. Mato Grosso encompasses three biomes, the Amazon, Cerrado, and Pantanal, which contribute to its high avian biodiversity [9].

Despite the significant diversity of haemosporidians, most existing data focus on Passeriformes, which account for 87% of the records in the Avian Malaria Initiative, MalAvi [3,10]. Because of the challenges in capturing non-passerine birds such as Strigiformes, Galliformes, Cariamiformes, and Falconiformes, considerable gaps remain in our understanding of parasitism in many non-passerine avian hosts. Wildlife hospitals, rehabilitation centers, and zoological institutions significantly contribute to enhancing our understanding of hemosporidian diversity within these often underrepresented avian taxa.

In this study, we investigated the occurrence of haemosporidian parasites in non-passerine birds in the state of Mato Grosso, Brazil. Samples were obtained from the Veterinary Hospital of the Federal University of Mato Grosso (HOVET/UFMT), which receives both free-living and captive birds for clinical care and treatment. The Wild Animal Sector of HOVET/UFMT regularly treats birds from various orders. We conducted morphological and molecular characterizations of *Plasmodium* and *Haemoproteus* parasites using microscopy, sequencing, and phylogenetic analyses. Research focusing on non-passerine birds from under-sampled regions such as this could provide valuable insights into the biodiversity of haemosporidians in Brazil.

## 2. Materials and Methods

### 2.1. Sample Collection and Study Area

All avian blood samples were obtained at the Veterinary Hospital of the Federal University of Mato Grosso (HOVET/UFMT), located in the municipality of Cuiabá, Brazil. Sampling was conducted during the routine treatment of wild animals between October 2021 and March 2024. The hospital receives birds from three main sources: free-living birds rescued by Brazilian environmental agencies (the Secretariat of State for the Environment—SEMA—and the Environmental Military Police of Mato Grosso), captive birds from the Center for Medicine and Research in Wild Animals (CEMPAS/UFMT), and pet birds brought in by the public.

**Figure 1:**
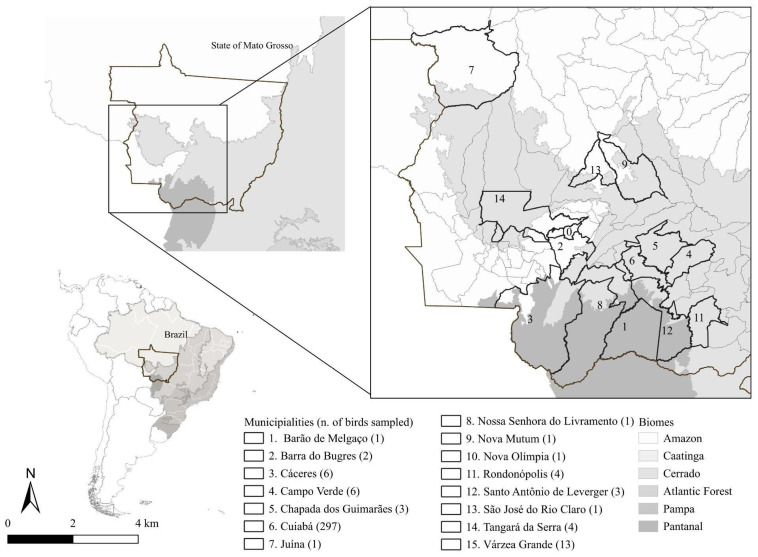
Map of Mato Grosso state showing municipalities where birds were screened for *Plasmodium* spp. and *Haemoproteus* spp. infections, based on microscopic examination and PCR analysis, from October 2021 to March 2024.

All birds sampled in this study originated from 15 municipalities in the state of Mato Grosso, Brazil (Figure 1). However, most birds sampled in this study originated from the Cerrado biome, with 297 of the 344 individuals (86.34%) collected in Cuiabá, a municipality characterized by a rainy season from October to March, a dry season from April to September, temperatures of 22–27 °C, and an average annual rainfall of ~1500 mm [11]. Only one bird (0.29%) was sampled in the municipality of Juína, within the Amazonia biome, characterized by a super-humid equatorial climate [12]. The remaining 46 birds (13.37%) were collected in municipalities within the Cerrado or in transition zones between the Cerrado and Pantanal biomes. Although the Pantanal is defined by its flood–drought cycle [13], its fauna and flora are similar to those of the Cerrado biome [14].

Blood was collected via jugular vein puncture or from another vein deemed more suitable for each species. An aliquot of each blood sample was stored in an EDTA (ethylenediaminetetraacetic acid) tube and frozen at −20 °C until DNA extraction. Additionally, blood smears were prepared using standard procedures and stained with Rosenfeld’s stain [15].

### 2.2. Microscopic Examination and Morphological Characterization

Blood smears were examined under a Leica DM500 optical microscope at a magnification of 400×. The length of each slide was measured during this process. To image the hemosporidians, an Olympus CX31 light microscope equipped with the Q-Color5 imaging system from Olympus (Tokyo, Japan) and QCapture Pro7 imaging software from QImaging (Surrey, BC, Canada) was used. Microphotographs taken during this examination were used to support the morphological characterization of the observed forms, according to the identification key [1]. Additionally, ImageJ software version 1.54 was employed to measure the parasites, including their structures and positioning within host cells, to aid in species identification [4,5]. Parasitemia was estimated by analyzing 200 microscopic fields at a magnification of 1000× [16]. Only regions of the smears without overlapping erythrocytes were examined, with each field containing approximately 100 cells. The parasitemia intensity was determined by counting the number of parasites per 20,000 total cells.

### 2.3. Molecular Detection of Plasmodium spp. and Haemoproteus spp.

To diagnose hemosporidians at the molecular level, DNA was extracted using the phenol–chloroform method, followed by isopropanol precipitation, as described by Sambrook and Russell [17]. A total of 20 μL of blood from each bird was used for this process. At the end of the extraction, DNA was eluted in 50 μL of Tris-EDTA (TE) buffer and stored at −20 °C. The extracted DNA was tested using conventional polymerase chain reaction (cPCR), following the methods described by Fallon et al. [18], to detect a 154-base-pair (bp) ribosomal RNA coding sequence within the mitochondrial DNA (mtDNA) of haemosporidians in the genera *Plasmodium* and *Haemoproteus*. For the PCR reactions, 2 µL of DNA were amplified with primers 343F (5′-GCTCACGCATCGCTTCT-3′) and 496R (5′-GACCGGTCATTTTCTTTG-3′) under thermocycling conditions based on the protocol described by Roos et al. [19]: initial denaturation for 2 min at 94 °C followed by 35 cycles with 1 min denaturation at 94 °C, 1 min annealing at 62 °C, and 1 min 10 s extension at 72 °C, with a final extension for 3 min at 72 °C.

All positive samples were subsequently subjected to nested PCR (nPCR) to amplify a 478 bp fragment of the mitochondrial cytochrome b (*cytb*) gene of *Plasmodium* and *Haemoproteus*, as described by Hellgren et al. [20]. For the first PCR reaction, 2 μL of DNA was combined with the primers HaemNFI (5′-CATATATTAAGAGAAITATGGAG-3′) and HaemNR3 (5′-ATAGAAAGATAAGAAATACCATTC-3′) under thermocycling conditions: 30 s at 94 °C, 30 s at 50 °C, and 45 s at 72 °C for 20 cycles. The samples were incubated before the cyclic reaction at 94 °C for 3 min and after the cyclic reaction at 72 °C for 10 min. In the second reaction, 1 μL of the amplified product from the first reaction was used together with the primers HaemF (5′-ATGGTGCTTTCGATATATGCATG-3′) and HaemR2 (5′-GCATTATCTGGATGTGATAATGGT-3′) under the same conditions as in the first reaction, but with 35 cycles. All PCRs used *Plasmodium gallinaceum* as the positive control and sterile ultrapure water as the negative control.

The nPCR-positive samples were purified following the protocol described by Sambrook and Russell [17] and sequenced using the dideoxynucleotide chain-termination method originally developed by Sanger et al. [21]. Sequencing was performed at the René Rachou Research Institute in Belo Horizonte, Minas Gerais, Brazil, using an ABI 3730 DNA Analyzer (Applied Biosystems, Waltham, MA, USA), a capillary electrophoresis-based automatic sequencer.

### 2.4. Phylogenetic Analysis

We edited and verified the quality of the recovered sequences using ChromasPro software (Technelysium Pty Ltd., South Brisbane, Australia). These sequences were compared with those available in GenBank (https://www.ncbi.nlm.nih.gov/genbank/, accessed on 13 September 2024) and the MalAvi database [6]. The sequences were aligned using MUSCLE in MEGA11 software [22]. A divergence of one or more nucleotides was considered sufficient to describe distinct *cytb* lineages [23].

The best-fit model for phylogenetic analysis was selected using IQ-TREE software [24], resulting in a partitioned model with the following schemes: partition 1—K3Pu+F+ASC+R2; partitions 2 and 3—GTR+F+ASC+R4. Model selection was based on the most appropriate nucleotide substitution rate for each codon partition. For phylogenetic inferences using Bayesian analysis, Mr.Bayes version 3.2.7 [25] was employed. Two Markov chains were run simultaneously for 1,000,000 generations, with samples taken every 1000 generations. The first 250,000 trees, representing 25% of the total, were discarded, and the remaining trees were used to calculate posterior probabilities. In this analysis, *Leucocytozoon cariamae* was used as an out-group [26].

## 3. Results

### 3.1. Occurrence of Haemosporidian Infection

Overall, 65 of the 344 birds (18.89%) tested positive for *Plasmodium* or *Haemoproteus* using at least one of three diagnostic techniques: blood smear, cPCR, and nPCR. Free-living birds comprised the majority of the sample, 61.63% (*n* = 212), while captive birds represented 38.37% (*n* = 132) (Table 1). Regarding the origin of the positive birds, 17.92% (*n* = 38) were wild and 20.45% (*n* = 27) were captive, indicating that captive birds exhibited a slightly higher infection rate in this study.

### 3.2. Molecular and Phylogenetic Analysis of Plasmodium spp.

**Figure 2:**
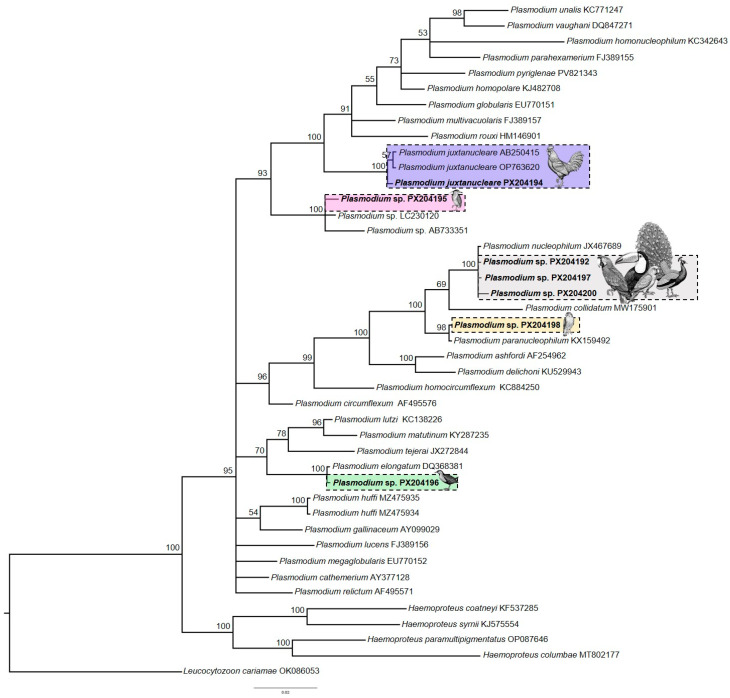
Bayesian phylogenetic inference based on 478 nucleotides of the cytochrome *b* gene. The *Plasmodium* sequences identified in this study are highlighted in bold, along with other *Plasmodium* sequences obtained from GenBank and MalAvi. *Leucocytozoon cariamae* CARCRI01 (accession No. OK086053) was used as the out-group.

Based on molecular data, we found 11 birds infected with *Plasmodium* lineages, nine of which yielded sequences of sufficient quality to achieve identity in GenBank. Three sequences showing more than 99% identity with *P. nucleophilum* were detected in four different host species: Red-and-green Macaw (*Ara chloropterus*), Indian Peafowl (*Pavo cristatus*), White-eyed Parakeet (*Psittacara leucophtalmus*), and Toco toucan (*Ramphastos toco*) (Table 2). In the phylogenetic reconstruction using Bayesian inference, these sequences clustered with haplotype JX467689, indicating that they belonged to the *P. nucleophilum* clade (Figure 2). No parasite erythrocytic forms were observed in any of the analyzed individuals.

We also detected two Roadside Hawks (*Rupornis magnirostris*) infected with lineages RUPMAGS-1335 and RUPMAGS-1328 (GenBank accession: PX204193 and PX204198), both of which showed 100% similarity to sequence KX159495 of *P. paranucleophilum*. Microscopic examination of blood smears revealed parasitemia levels of 0.005% and 0.015% and confirmed the presence of gametocytes, meronts, and trophozoites (see Supplementary Figure S1). *P. nucleophilum* and *P. paranucleophilum* are closely related phylogenetically, forming a subclade with *P. collidatum* (Figure 2).

Sequences of *P. juxtanucleare* (PX204194) and *P. elongatum* were identified in the Domestic Chicken (*Gallus gallus*) and Ash-throated Crake (*Mustelirallus albicollis*). Although no blood stages of the parasite were observed in the smears, the morphospecies was confirmed based on the recovered sequences, which showed 100% similarity to previously deposited database entries. Another sequence was recovered from a Black-Crowned Night-Heron (*Nycticorax nycticorax*) individual (PX204195), which clustered phylogenetically within a clade of lineages not yet associated with any described morphospecies (Figure 2). Blood smear analysis revealed 0.12% parasitemia, and only gametocytes were observed, limiting the morphological characterization of this parasite (see Supplementary Figure S1).

### 3.3. Molecular, Phylogenetic, and Morphological Analysis of Haemoproteus spp.

**Figure 3:**
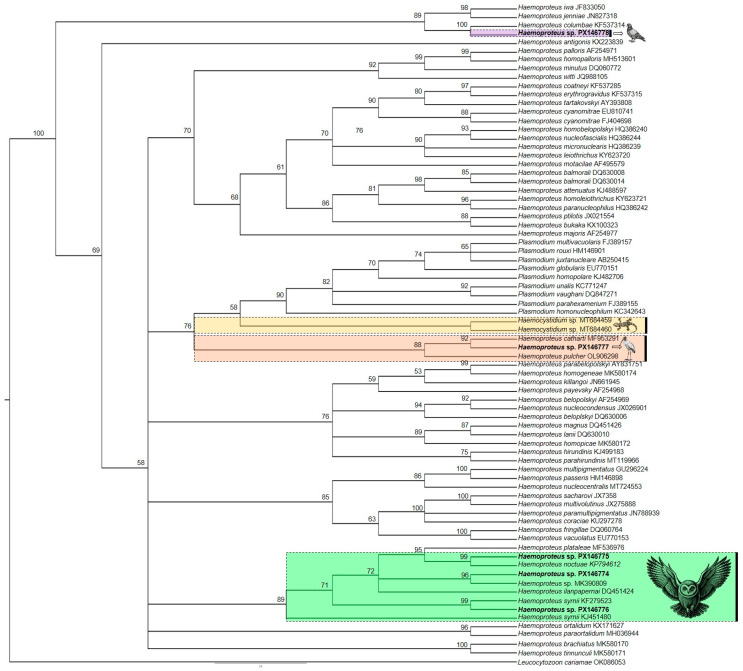
Bayesian phylogenetic inference based on 478 nucleotides of the cytochrome *b* gene. The *Haemoproteus* sequences identified in this study are highlighted in bold, alongside other *Haemoproteus* sequences sourced from GenBank and MalAvi. *Leucocytozoon cariamae* CARCRI01 (accession No. OK086053) serves as the out-group.

**Figure 4:**
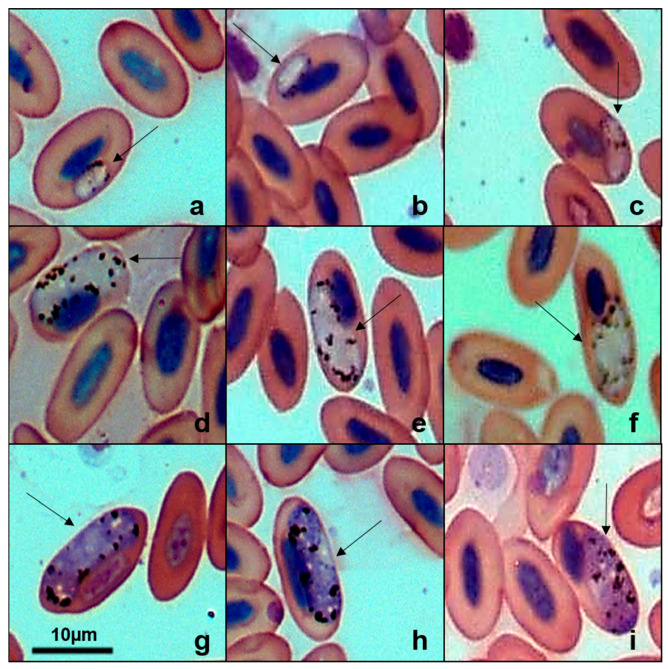
Plate exhibiting gametocytes of *Haemoproteus* sp. (GenBank accession: PX146777) from the blood smear of the Wood Stork (*Mycteria americana*). (**a**–**c**): young gametocytes. (**d**–**f**): microgametocytes. (**g**–**i**): macrogametocytes. The arrows indicate the different parasitic forms inside the red blood cells. Scale bar = 10 μm.

Five lineages of *Haemoproteus* were found in 344 birds (Table 2), with four new lineages deposited in GenBank: Burrowing Owl (*Athene cunicularia*) (PX146775), Striped Owl (*Asio clamator*) (PX146776), Picazuro Pigeon (*Patagioenas picazuro*) (PX146778), and American Barn Owl (*Tyto furcata*) (PX146774). The unpublished haplotype identified in *P. picazuro* showed 98.54% sequence similarity with lineage MN065207 previously reported in Rock Pigeons (*Columba livia*). Our phylogenetic analysis (Figure 3) clustered these two haplotypes, but it was not possible to determine the morphospecies associated with this lineage. From Wood Stork (*Mycteria americana*), we recovered the lineage MYCAMES-1685 (GenBank accession: PX146777), which showed 100% similarity with lineage MYCAMH1 (GenBank accession: JX546141). Phylogenetically, this sequence clustered in a distinct clade, separate from other *Haemoproteus* species, including the morphospecies *Haemoproteus catharti* and *H. pulcher*. Examination of blood smears from M. americana revealed gametocytes infecting erythrocytes (Figure 4) with a parasitemia rate of 0.16%. However, we were unable to obtain a detailed morphological description of the parasite.

**Figure 5:**
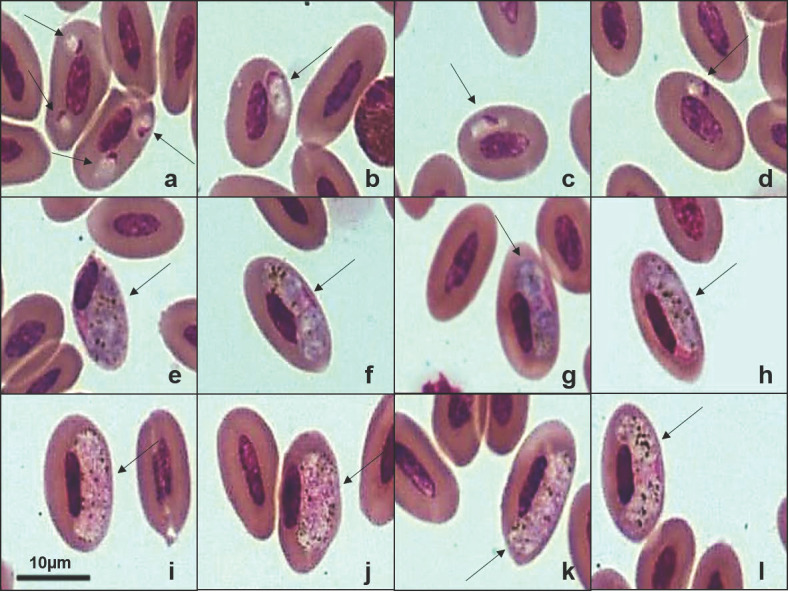
Plate exhibiting gametocytes of *Haemoproteus* sp. (PX146774) from the blood smear of the American Barn Owl (*Tyto furcata*). (**a**–**d**): young gametocytes. (**e**–**h**): macrogametocytes. (**i**,**j**,**h**,**l**): microgametocytes. The arrows indicate the different parasitic forms inside the red blood cells. Scale bar = 10 μm.

The new lineages detected in *A. cunicularia* and *A. clamator* were more than 99% similar to *Haemoproteus noctuae* and *Haemoproteus syrnii* (Table 2). However, it was not possible to confirm the morphospecies based on erythrocytic forms. We also identified a new lineage of *Haemoproteus* sp. (TYTFURS-1384, PX146774) infecting *T. furcata*, which showed 98.74% sequence identity with lineage MK390809 previously reported in *Tyto alba* [27]. The host presented gametocytes infecting erythrocytes in blood smears, with a parasitemia rate of 0.2%. Morphologically, these parasites exhibited young oval gametocytes that typically contacted the erythrocyte nucleus and were located in a polar or subpolar position relative to the nucleus (Figure 5a–d). We observed erythrocytes infected with two or more young gametocytes (Figure 5a). Mature gametocytes displayed the classical morphological dimorphism [1] with macrogametocytes having a dense nucleus and more intensely stained cytoplasm compared to microgametocytes. Both microgametocytes and macrogametocytes were elongated and positioned laterally to the erythrocyte nucleus, making contact with the nucleus and cell envelope. The nuclei of the gametocytes were located in distinct areas within the cytoplasm, appearing in both polar (Figure 5h) and central (Figure 5f,l) regions of the parasite. Additionally, malaria pigment and volutin granules were irregularly distributed in both microgametocytes and macrogametocytes. This new haplotype, TYTFURS-1384, is closely related to *Haemoproteus ilanpapernai* [28] but exhibits distinct morphological characteristics. All *Haemoproteus* lineages were recovered from the Strigiformes cluster within the same major clade. However, three well-supported subclades separated the morphospecies: *H. syrnii*, *H. noctuae*, and *H. ilanpapernai*. These findings suggest that the parasite infecting *T. furcata* represents a distinct *Haemoproteus* species.

## 4. Discussion

Most studies on avian hemosporidians have focused on Passeriformes, which are more easily sampled using relatively simple methods such as mist nets [7,29]. Consequently, few studies have encompassed the wide diversity of non-passerine bird orders and species. This study addresses this research gap by examining a diverse range of non-passerine bird species. It revealed a prevalence of haemosporidian infections of 18.89%, with 344 individuals across 60 species and 16 orders in Mato Grosso State, Brazil testing positive. In a related study, Chagas et al. [30] reported a similar prevalence of 18% in free-living birds using PCR-based diagnostic methods, a result very similar to that observed in our wild birds (17.92%). Furthermore, Chagas et al. [31] conducted an extensive study on non-passerines in captivity in São Paulo, including 677 birds from 17 orders and 122 species, and found an infection rate of 12.6% for *Plasmodium* spp. and/or *Haemoproteus* spp., therefore lower than the 20.45% infection rate recorded for the captive birds in this study.

To date, no other study in Brazil has examined such a diverse and extensive sample of non-passerine bird species. For example, Belo et al. [32] found that 36% (46 of 127) of avian malaria cases were restricted to captive parrots from three Brazilian zoos. Morel et al. [33] reported an infection rate of 27% (56 of 206) in free-living raptors, whereas Vanstreels et al. [34] detected a prevalence of 64.3% (18 of 28) in captive penguins undergoing rehabilitation. Anjos et al. [35] analyzed 399 birds, most of which belonged to Passeriformes (362 out of 399; 90.7%), and identified only one infected individual among the non-passerines (Apodiformes). In contrast, our study found haemosporidian infections (*Haemoproteus* and *Plasmodium*) across 16 non-passerine orders, highlighting the broader host range and ecological significance of these parasites. The data from this bird group in the present study, which presented limited information in the literature, provide relevant insights into haemosporids; however, the lack of morphological data associated with molecular data for some individuals represents a gap that warrants further investigation in future studies.

Detection and characterization of these parasites, as well as understanding their transmission dynamics, are essential to minimizing risks to biodiversity, particularly in species subject to illegal trafficking or facing extinction risk. According to the International Union for Conservation of Nature (IUCN), our sampling included several threatened bird species [36]. Species listed as near threatened (NT), including *Harpia harpyja*, *Alipiopsitta xanthops*, *Primolius maracana*, and *Rhea americana*, as well as one vulnerable (VU) species, *Anodorhynchus hyacinthinus*, were sampled for the present study. Although only *A. xanthops* tested positive for haemosporidians, continued monitoring of these taxa remains crucial for conservation efforts. A large proportion of the IUCN-listed species in our dataset belong to Psittaciformes, a group commonly observed in the wild, maintained in captivity (e.g., zoos), or involved in illegal trade networks [37]. Despite the generally low prevalence of haemosporidian infection in psittaciform birds, these parasites can be pathogenic to them [38,39]. Moreover, environmental and ecological changes may alter parasite–host interactions, potentially affecting bird communities [40].

Over the years, avian *Plasmodium* species have been recognized as generalist parasites [3]. For example, *P. nucleophilum* is a cosmopolitan species found in various bird hosts [31]. Using molecular analyses, we identified three distinct sequences from four different hosts that exhibited high similarity and clustered phylogenetically with these morphospecies. However, the lack of morphological data prevented us from confirming whether these birds could serve as competent hosts for the development of *P. nucleophilum*. Nonetheless, future studies should investigate whether the host species (*Ara chloropterus*, *Pavo cristatus*, *Psittacara leucophthalmus*, and *Ramphastos toco*) act as potential reservoirs for the spread of this parasite. *A. chloropterus* and *P. leucophthalmus* are Neotropical species endemic to the Americas and are frequently targeted in the illegal wildlife trade [41,42,43], which could facilitate parasite transmission. Although *P. cristatus* is native to India, it is an ornamental species commonly traded and maintained in captivity worldwide [44], potentially contributing to the global distribution of *P. nucleophilum*. To our knowledge, this is the first report of *Plasmodium* spp. in *A. chloropterus*. In the case of *P. leucophthalmus*, although a report of haemosporidian infection exists, it was not determined whether *Plasmodium* or *Haemoproteus* caused the infection. Therefore, this represents the first report of *Plasmodium* spp. in this host.

Another cosmopolitan and generalist parasite is *P. elongatum*, a morphospecies that includes several misidentified lineages [4]. In our study, we recovered a lineage identical to sequence DQ368381, which is widely accepted as *P. elongatum* [4]. Although no morphological data were obtained, *M. albicollis* should be further investigated as a potential host for this parasite, and this may represent the first report of a hemosporidian infection in this host. We also detected *R. magnirostris* infections in a lineage associated with the morphospecies *P. paranucleophilum*. Tostes et al. [45] described *P. paranucleophilum* in various avian hosts, including *R. magnirostris*, suggesting a generalist profile. Our findings reinforce the role of *R. magnirostris* as a competent host of this parasite. In contrast to the generalist species mentioned earlier, we identified a sequence of *P. juxtanucleare* from its typical host, *G. gallus*, indicating that this parasite has a more specialized host preference. Despite the strong association between host and parasite, Ferreira et al. [46] reported instances of *P. juxtanucleare* infecting passerine birds. Given the pathogenic nature of this species, such spillover events are ecologically significant and may lead to increased mortality in wild bird populations.

Additionally, we detected lineage PX204195 in *N. nycticorax* individuals. Morphologically, this haplotype has not yet been characterized, nor have other sequences clustered within the same clade. These findings indicate the presence of a previously undescribed *Plasmodium* species or a lineage of a known morphospecies that has not yet been linked to molecular data. It is worth noting that only about half of all *Plasmodium* morphospecies are currently associated with genetic lineages [3].

In our study, haplotype PX146775 was recorded from *A. cunicularia* and showed 99.58% identity with *H. noctuae* (GenBank accession: ON932226). An individual of *A. clamator* was infected with sequence PX146776, which exhibited 98.74% identity with the *H. syrnii* sequence (GenBank accession: KJ575554). Phylogenetically, both sequences clustered within the clade represented by *H. syrnii* and *H. noctuae*, suggesting that despite the absence of morphological data, the parasites likely belong to this species complex.

Historically, more than ten *Haemoproteus* species infecting Strigiformes have been described [47]. To “clarify this chaotic situation,” Bishop and Bennett [47] redescribed *H. syrnii*, *H. noctuae*, and 11 other *Haemoproteus* species that infect owls, highlighting the taxonomic confusion within this group. Subsequent studies synonymized many of these species under *H. syrnii* and *H. noctuae* due to their high morphological similarity [1,5], resulting in the formation of a species complex. The first molecular sequence of *H. syrnii* was obtained in 2006, which matched the corresponding morphospecies [3], providing a genetic reference for this taxonomically complex group.

Approximately 100 years after its original description, Karadjian et al. [48] reopened the taxonomic discussion of *H. syrnii* and other *Haemoproteus* parasites that can infect owls. The subsequent description of *H. ilanpapernai* [28], associated with sequence DQ451424 (lineage STSEL01), suggested a possibly greater diversity of species infecting Strigiformes beyond *H. syrnii* and *H. noctuae*. Currently, the lineages OTSCO05, STAL02, and CULKIB01 (with respective GenBank accession numbers: KJ451480, KF279523, and KP794611) are commonly used as molecular barcodes for identifying *H. syrnii* [5].

Our study also reported, for the first time, the occurrence of a *Haemoproteus* parasite that infects *Tyto furcata*. The gametocytes observed in blood smears from this host exhibited morphological characteristics distinct from those of *H. syrnii* and *H. noctuae*. Notably, the parasite found in our study was shorter in length and contained fewer malarial pigments in the cytoplasm than the two well-characterized species. The gametocytes displace the erythrocyte nucleus without completely encircling it, which distinguishes this parasite from *H. noctuae*. Furthermore, immature gametocytes lacked volutin granules, a feature that Mayer [49] originally noted as prominent in *H. syrnii*. *H. ilanpapernai* gametocytes are smaller than those observed in *T. furcata* and do not displace the erythrocyte nucleus.

The sequence recovered from *T. furcata* corresponded to a new lineage, PX146774, which exhibited more than 2.72% genetic divergence compared to sequences attributed to *H. syrnii*, 2.52% to *H. noctuae*, and 2.22% compared to *H. ilanpapernai*. Furthermore, phylogenetic analyses showed that this new lineage of *H. ilanpapernai* formed a distinct clade separate from the haplotypes of *H. syrnii* and *H. noctuae*. Barino et al. [50] conducted a phylogenetic study using different sequences identified as *H. syrnii* and observed divergence among these lineages, leading to their classification into distinct clades and revealing the paraphyly of the species. Although they did not identify significant morphological differences between the recovered parasites and *H. syrnii*, they suggested the possible presence of a cryptic species. Giorgiadis et al. [51] evaluated captive Strigiformes in France and identified three haplotypes infecting these birds, corresponding to two distinct morphospecies. Haplotypes A and C were similar to *H. syrnii*, whereas haplotype B represented a morphologically and genetically distinct species unrelated to other *Haemoproteus* parasites typically found in Strigiformes.

Our phylogenetic analyses corroborated that the different *Haemoproteus* morphospecies infecting owl clusters form separate clades, as evidenced by the haplotypes of *H. syrnii*, *H. noctuae*, and *H. ilanpapernai*. Parasites infecting Strigiformes may have a complex history, and recent data have indicated a greater diversity of species than the traditionally recognized *H. syrnii* and *H. noctuae*. Although our data do not support the presence of a new species, they do indicate that the differences among *Haemoproteus* species infecting Strigiformes warrant further investigation. *Haemoproteus* parasites are classified, based on vector transmission, into two subgenera: *Parahamoproteus* and *Haemoproteus* [5]. In general, owls are nocturnal birds, and this could influence the invertebrate host to which these birds are exposed [52]. Due to that, the vectors for those parasites from owls may be distinct from those that infect diurnal animals.

Another finding was the report of a new lineage infecting *P. picazuro*, which showed 98.74% sequence identity to *H. columbae* (GenBank accession: LC606013). We could not perform a morphological evaluation. Despite the genetic differences between our haplotype and the sequences available in the database, sequence PX146778 clustered with *H. columbae* in the phylogenetic analysis. *H. columbae* was originally described by Kruse [53] and was previously believed to exclusively infect the domestic pigeon, *Columba livia* [54]. However, subsequent studies have demonstrated that this species can infect several Columbiformes [1,55]. As there are no previous records of *P. picazuro* infected by *H. columbae* or any other haemosporidian parasite, future studies should investigate this bird as a potential new host for the parasite.

We also recovered a sequence of *Haemoproteus* sp., identified as PX146777, which showed 100% identity with the lineage JX546141, as previously reported by Villar et al. [56]. In both studies, the haplotype was recovered from *M. americana*. Phylogenetic analysis revealed that this haplotype formed a clade with *H. catharti* and *H. pulcher*. Genetically, the PX146777 sequence exhibited 2.53% and 5.47% divergence from *H. catharti* and *H. pulcher*, respectively. This level of divergence indicates that the sequence from *M. americana* represents a new species. However, it was not possible to establish a link between the PX146777 lineage and the parasite morphology. Phylogenetically, these lineages clustered in a distinct subclade closely related to *Hemocystidium* and *Plasmodium* parasites. Studying the morphospecies and lineages of this clade is important for understanding the evolutionary relationships among haemosporidians.

## 5. Conclusions

Non-passerine birds exhibit remarkable diversity in avian haemosporidians. However, due to the inherent challenges associated with sampling these hosts, both morphological and genetic data regarding hemosporidian parasites remain limited. It is likely that many undescribed species within the genera *Plasmodium* and *Haemoproteus* exist, as suggested by the identification of the PX146774 lineage in *T. furcata*. Consequently, these avian groups represent significant targets for future research aimed at uncovering the evolutionary history of haemosporidians and their host–parasite interactions. Our study introduces new lineages and provides important insights into the biodiversity of hemosporidian parasites, further highlighting the significance of non-passerine birds in advancing this field.

## Figures and Tables

**Table 1:** Origin of bird species treated in the Wild Animal Sector of the Veterinary Hospital at the Federal University of Mato Grosso, Cuiabá, MT, from October 2021 to March 2024, and screened for the morphological and/or molecular detection of *Plasmodium* spp. and *Haemoproteus* spp.

Order (No. of Birds) Species (No. of Birds)	Common Name (Degree of Threat ^a^)	No. of Birds Tested in Blood Smear	No. of Birds Tested in cPCR and nPCR	Origin ^b^ (No. of Birds)	Municipality ^c^ (No. of Birds)
Accipitriformes (41)					
*Gampsonyx swainsonii* (1)	Pearl Kite (LC)	1	ne ^d^	FL (1)	6 (1)
*Harpia harpija* (1)	Harpy Eagle (NT)	1	ne	FL (1)	7 (1)
*Heterospizias meridionalis* (2)	Savanna Hawk (LC)	1	2	FL (2)	3 (2)
*Ictinia plumbea* (3)	Plumbeous Kite (LC)	3	ne	FL (3)	6 (2), 13 (1)
*Pandion haliaetus* (1)	Osprey (LC)	1	ne	FL (1)	6 (1)
*Rupornis magnirostris* (32)	Roadside Hawk (LC)	31	14	FL (18), C (14)	1 (1), 4 (1), 6 (28), 15 (2)
*Urubitinga urubutinga* (1)	Great Black Hawk (LC)	1	ne	FL (1)	6 (1)
Anseriformes (6)					
*Anser cygnoides* (1)	Swan Goose (EN)	1	1	C (1)	6 (1)
*Cairina moschata* (3)	Muscovy Duck (LC)	ne	3	C (3)	6 (3)
*Chauna torquata* (2)	Southern Screamer (LC)	ne	2	C (2)	6 (2)
Cariamiformes (9)					
*Cariama cristata* (9)	Red-legged Seriema (LC)	4	8	FL (7), C (2)	6 (7), 8 (1), 11 (1)
Cathartiformes (1)					
*Coragyps atratus* (1)	Black Vulture (LC)	1	1	FL (1)	6 (1)
Ciconiiformes (5)					
*Jabiru mycteria* (4)	Jabiru (LC)	1	4	FL (4)	6 (4)
*Mycteria americana* (1)	Wood Stork (LC)	1	1	FL (1)	10 (1)
Columbiformes (3)					
*Columba livia* (1)	Rock Pigeon (LC)	ne	1	FL (1)	6 (1)
*Geotrygon montana* (1)	Ruddy Quail-Dove (LC)	ne	1	FL (1)	6 (1)
*Patagioenas picazuro* (1)	Picazuro Pigeon (LC)	1	1	FL (1)	14 (1)
Cuculiformes (1)					
*Piaya cayana* (1)	Squirrel Cuckoo (LC)	1	1	FL (1)	6 (1)
Falconiformes (18)					
*Caracara Plancus* (12)	Southern Caracara (LC)	12	3	FL (5), C (7)	6 (12)
*Falco femoralis* (2)	Aplomado Falcon (LC)	1	1	C (2)	6 (2)
*Falco sparverius* (1)	American Kestrel (LC)	1	ne	C (1)	6 (1)
*Herpetotheres cachinnans* (1)	Laughing Falcon (LC)	1	1	FL (1)	6 (1)
*Milvago chimachima* (1)	Yellow-headed Caracara (LC)	1	1	FL (1)	6 (1)
*Micrastur semitorquatus* (1)	Collared Forest-Falcon (LC)	1	1	FL (1)	3 (1)
Galiformes (9)					
*Gallus gallus* (7)	Domestic chicken (LC)	7	4	C (7)	6 (7)
*Ortalis conicollis* (1)	Chaco chachalaca (LC)	1	1	FL (1)	15 (1)
*Pavo cristatus* (1)	Indian Peafowl (LC)	1	1	C (1)	6 (1)
Gruiformes (2)					
*Mustelirallus albicollis* (1)	Ash-throated Crake (LC)	1	1	FL (1)	6 (1)
*Porphyrio martinica* (1)	Purple Gallinule (LC)	ne	2	FL (1)	6 (1)
Nyctibiiformes (5)					
*Nyctibius grandis* (1)	Great Potoo (LC)	1	1	FL (1)	6 (1)
*Nyctibius griseus* (4)	Common Potoo (LC)	4	4	FL (4)	6 (4)
Pelecaniformes (3)					
*Bulbucus ibis* (1)	Cattle Egret (LC)	1	1	FL (1)	6 (1)
*Egretta thula* (1)	Snowy Egret (LC)	1	1	FL (1)	6 (1)
*Nycticorax nycticorax* (1)	Black-crowned Night-Heron (LC)	1	1	FL (1)	6 (1)
Piciformes (13)					
*Pteroglossus castanotis* (1)	Chestnut-eared Aracari (LC)	1	ne	FL (1)	5 (1)
*Ramphastos toco* (11)	Toco toucan (LC)	ne	11	FL (3), C (8)	6 (11)
*Veniliornis affinis* (1)	Red-stained Woodpecker (LC)	1	1	FL (1)	6 (1)
Psittaciformes (186)					
*Agapornis roseicolis* (2)	Agapornis (LC)	ne	2	C (2)	6 (2)
*Alipiopsitta xanthops* (3)	Yellow-faced Parrot (NT)	1	3	FL (1), C (2)	6 (3)
*Amazona aestiva* (9)	Turquoise-fronted Parrot (LC)	4	8	FL (4), C (5)	6 (9)
*Amazona amazonica* (17)	Orange-winged Parrot (LC)	5	16	FL (4), C (13)	6 (17)
*Amazona ochrocephala* (2)	Yellow-crowned Parrot (LC)	1	1	FL (1), C (1)	6 (2)
*Anodorhynchus hyacinthinus* (3)	Hyacinth Macaw (VU)	1	3	FL (3)	4 (1), 6 (2)
*Ara ararauna* (95)	Blue-and-yellow Macaw (LC)	73	56	FL (86), C (9)	3 (2), 4 (4), 5 (1), 6 (82), 11(3), 14 (1), 15 (2)
*Ara chloropterus* (9)	Red-and-green Macaw (LC)	4	9	FL (6), C (3)	2 (1), 6 (5), 15 (3)
*Ara macao* (2)	Scarlet Macaw (LC)	ne	2	FL (2)	6 (2)
*Ara severus* (1)	Chestnut-fronted Macaw (LC)	1	1	C (1)	6 (1)
*Brotogeris chiriri* (8)	Yellow-chevroned Parakeet (LC)	4	7	FL (7), C (1)	6 (7), 15 (1)
*Diopsittaca nobilis* (5)	Red-shouldered Macaw (LC)	2	4	FL (5)	6 (3), 15 (2)
*Eupsittula aurea* (9)	Peach-fronted Parakeet (LC)	4	8	FL (7), C (2)	5 (1), 6 (8)
*Nymphicus hollandicus* (15)	Cockatiel (LC)	14	15	C (15)	6 (14), 15 (1)
*Primolius auricolis* (2)	Yellow-collared Macaw (LC)	1	2	FL (1), C (1)	6 (2)
*Primolius maracana* (2)	Blue-winged Macaw Kite (NT)	1	1	FL (1), C (1)	6 (2)
*Psittacara leucophtalmus* (2)	White-eyed Parakeet (LC)	2	2	FL (1), C (1)	6 (2)
Rheiformes (2)					
*Rhea americana* (2)	Greater Rhea (NT)	ne	2	C (2)	6 (2)
Strigiformes (40)					
*Asio clamator* (5)	Spided Owl (LC)	4	3	FL (2), C (3)	6 (5)
*Athene cunicularia* (10)	Burrowing Owl (LC)	9	6	FL (6), C (4)	6 (8), 12 (2)
*Glaucidium brasilianum* (4)	Ferruginous Pygmy-Owl (LC)	4	1	FL (1), C (3)	6 (2), 14 (2)
*Megascops choliba* (7)	Tropical Screech-Owl (LC)	7	4	FL (2), C (5)	2 (1), 6 (5), 12 (1)
*Tyto furcata* (14)	American Barn Owl (LC)	13	9	FL (4), C (10)	3 (1), 6 (11), 9 (1), 15 (1)
Total (344)		241	240	FL (212), C (132)	1 (1), 2 (2), 3 (6), 4 (6), 5 (3), 6 (297), 7 (1), 8 (1), 9 (1), 10 (1), 11 (4), 12 (3), 13 (1), 14 (4), 15 (13)

^a^ Degree of threat according to the Red List of The International Union for Conservation of Nature’s (IUCN) https://www.iucnredlist.org/en, accessed on 10 September 2025): NE: not evaluated; DD: data deficient; LC: least concern; NT: near threatened; VU: vulnerable; EN: endangered; CR: critically endangered; EW: extinct in the wild; EX: extinct. ^b^ Origin: free-living (FL), captivity (C). ^c^ Municipality: 1. Barão do Melgaço, 2. Barra dos Bugres, 3. Cáceres, 4. Campo Verde, 5. Chapada dos Guimarães, 6. Cuiabá, 7. Juína, 8. Nossa Senhora do Livramento, 9. Nova Mutum, 10. Nova Olímpia, 11. Rondonópolis, 12. Santo Antônio do Leverger, 13. São José do Rio Claro,14. Tangará da Serra, 15. Várzea Grande. ^d^ ne: not evaluated due to absence of the sample (blood smear or whole blood).

**Table 2:** Positive birds of the hospital routine in the Wild Animal Sector at the Veterinary Hospital of the Federal University of Mato Grosso, Cuiabá, MT, from October 2021 to March 2024, submitted to the morphological and/or molecular detection of *Plasmodium* spp. and *Haemoproteus* spp.

Order Species (No. of Birds)	No. of Birds with Positive Blood Smears/Total Blood Smears Tested (%) (Parasitemia per Individual %)	No. of Birds Testing Positive in cPCR/Total n. of Birds Tested in cPCR (%)	No. of Birds Testing Positive in nPCR/Total No. of Birds Tested in nPCR (%)	No. of Sequences Obtained	% Similarity of Genus/Species of Hemosporid (GenBank Access Code)/Lineage—per Individual	Origin/City of Origin of Birds with Sequences Recovered—per Individual ^ab^
Accipitriformes						
*Heterospizias meridionalis* (2)	0/1	1/2 (50)	0/2 (0)	0		
*Rupornis magnirostris* (32)	2/31 (6.4) (0.005; 0.015)	5/14 (35.7)	3/14 (21.4)	2	100% *Plasmodium paranucleophilum* (PX204193; PX204198)/CPCT5 7 ^d,e^	FL/6; FL/6
Cariamiformes						
*Cariama cristata* (9)	1/4 (25) (0.12)	2/8 (25)	2/8 (25)	0		
Ciconiiformes						
*Jabiru mycteria* (4)	0/1	2/4 (50)	2/4 (50)	0		
*Mycteria americana* (1)	1/1 (100) (0.16)	1/1 (100)	1/1 (100)	1	100% *Haemoproteus* sp. (PX146777)/BULIBH1	FL/10
Columbiformes						
*Patagioenas picazuro* (1)	1/1 (100) (0.12)	1/1 (0)	1/1 (100)	1	98.54% *Haemoproteus columbae* (PX146778)/PATPIC1061328 ^e^	FL/14
Falconiformes						
*Caracara plancus* (12)	3/12 (25) (0.005; 0.085; 0.15)	1/3 (33.3)	0/3 (0)	0		
Galliformes						
*Gallus gallus* (7)	1/7 (14.3) (0.005)	1/4 (25)	1/4 (25)	1	100% *Plasmodium juxtanucleare* (PX204194)/Pj221	C/6
*Pavo cristatus* (1)	ne ^c^	1/1 (100)	1/1 (100)	1	99.79% *Plasmodium nucleophilum* (PX204197)/PAVCRI1088056 ^e^	
Gruiformes						
*Mustelirallus albicollis* (1)	1/1 (100) (0.02)	1/1 (100)	1/1 (100)	1	100% *Plasmodium elongatum* (PX204196)/MF101820	FL/6
*Porphyrio martinica* (1)	ne	1/1 (100)	1/1 (100)			
Pelecaniformes						
*Nycticorax nycticorax* (1)	1/1 (100) (0.12)	1/1 (100)	1/1 (100)	1	100% *Plasmodium* sp. (PX204195)/CETASFLO-R0263	FL/6
Piciformes						
*Ramphastos toco* (11)	ne	4/11 (36.4)	4/11 (36.4)	1	100% *Plasmodium* sp. (PX204199)/GAL-2012	C/6
Psittaciformes						
*Agapornis roseicolis* (2)	ne	0/2	2/2 (100)	0		
*Alipiopsitta xanthops* (3)	0/1	2/3 (66.7)	2/3 (66.7)	0		
*Amazona aestiva* (9)	0/4	3/8 (37.5)	0/8	0		
*Amazona amazonica* (17)	0/5	1/16 (6.2)	0/16	0		
*Ara ararauna* (95)	1/73 (1.4) (0.045)	7/55 (12.7)	3/55 (5.4)	0		
*Ara chloropterus* (9)	0/4	1/9 (11.1)	1/9 (11.1)	1	99.37% *Plasmodium nucleophilum* (PX204200)/ARACLOS-1657 ^e^	FL/2
*Brotogeris chiriri* (8)	0/4)	2/7 (28.6)	0/7	0		
*Diopsittaca nobilis* (5)	0/2	1/4 (25)	0/4	0		
*Eupsittula aurea* (9)	0/4	1/8 (12.5)	0/8	0		
*Nymphicus hollandicus* (15)	0/14	3/15 (20)	0/15	0		
*Psittacara leucophtalmus* (2)	1/2 (50) (0.005)	1/2 (50)	1/2 (50)	1	100% *Plasmodium nucleophilum* (PX204192)/RVCT27	C/6
Strigiformes						
*Asio clamator* (5)	4/4 (100) (0.525; 0.270; 0.180; 0.085)	2/3 (66.7)	2/3 (66.7)	1	99.16% *Haemoproteus syrnii* (PX146776)/ASICLAS-1682 ^e^	FL/6
*Athene cunicularia* (10)	1/9 (11.1) (0.015)	4/6 (66.7)	1/6 (16.7)	1	99.58% *Haemoproteus noctuae* (PX146775)/ATHCUNS-1386 ^e^	FL/6
*Glaucidium brasilianum* (4)	0/4 (0)	1/1 (100)	0/1	0		
*Megascops choliba* (7)	4/7 (57.1) (0.01; 0.105; 0.140; 0.105)	0/4	0/4	0		
*Tyto furcata* (14)	3/13 (23.1) (0.2; 0.125; 0.36)	3/9 (33.3)	1/9 (11.1)	1	98.12% *Haemoproteus plataleae* (PX146774)/TYTFURCS-1384 ^e^	FL/9
Total (%)	25/241 (10.37)	53/240 (22.08)	30/240 (12.5)	14		

^a^ Origin: free-living (FL) and captivity (C). ^b^ The municipality number is depicted in Figure 1. ^c^ ne: not evaluated due to absence of the sample (blood smear or whole blood). ^d^ Two individuals of the same bird species harbored identical lineages. ^e^ NL: new lineage.

## Data Availability

The sequences assemblies were deposited at GenBank repository https://www.ncbi.nlm.nih.gov/genbank/, accessed on 13 September 2024.

## Supplementary Materials

The following supporting information can be downloaded at https://www.mdpi.com/article/10.3390/pathogens14121286/s1, Figure S1: Plates containing parasites from different hosts. *Plasmodium paranucleophilum* (lineage CPCT57) from a Roadside Hawk (*Rupornis magnirostris*), (a): trophozoite; (b,c): meront; (d): gametocyte. *Plasmodium* sp. (lineage CETASLO-R0263) from a Black-crowned Night Heron (*Nycticorax nycticorax*); (e,f): trophozoites; (g): microgametocytes; (h): microgametocytes. *Haemoproteus syrnii* (lineage ASICLAS-1682) was isolated from the blood smear of the Striped Owl (*Asio clamator*). (i,j): macrogametocytes. (k,l): microgametocytes. The arrows indicate the different parasitic forms inside the red blood cells. Scale bar = 10 μm.
