# Supplementary figures and images for: Unraveling the Diversity of Haemosporidians in Brazilian Non-Passerine Birds: Insights from Midwestern Brazil
Source: Pathogens. 2025 Dec 13;14(12):1286. doi: 10.3390/pathogens14121286 (PMC12736227; doi:10.3390/pathogens14121286)

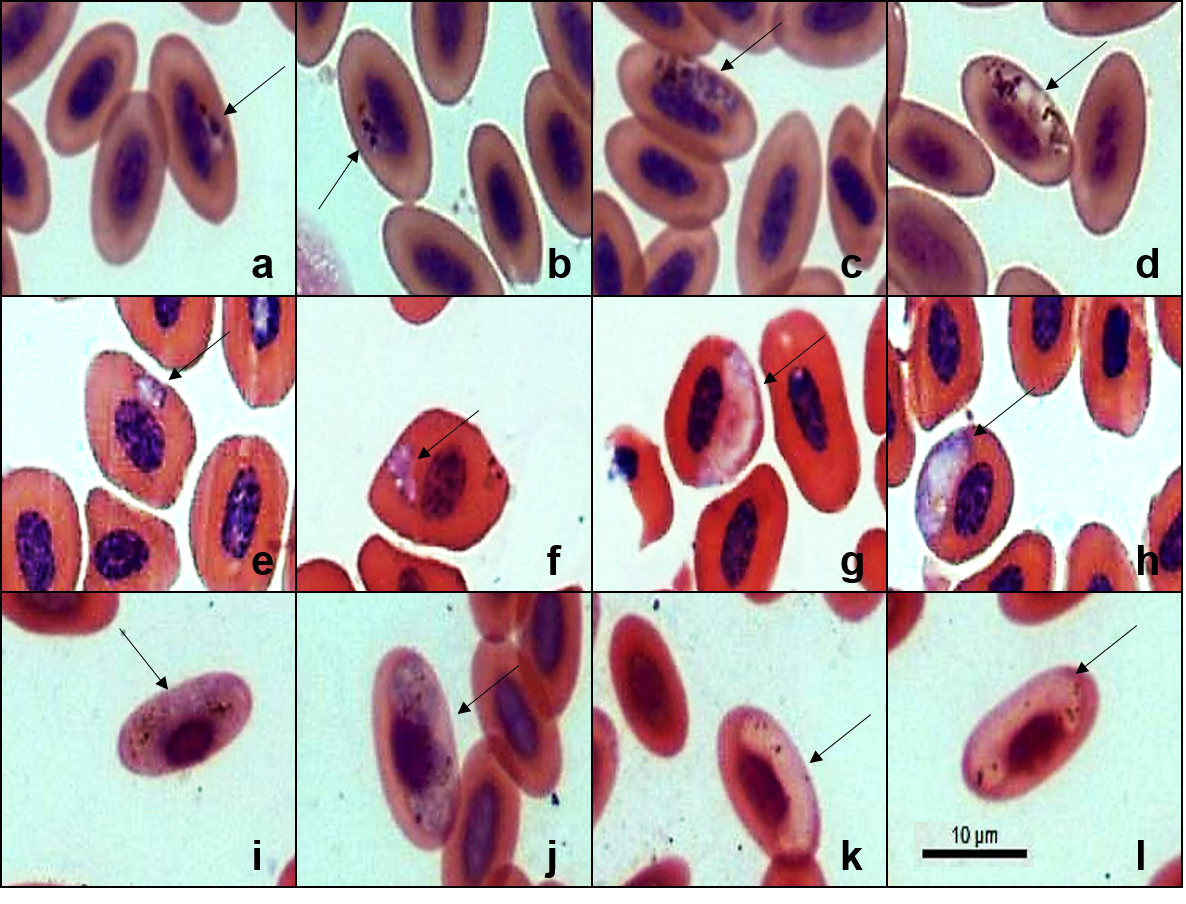

Supplement: Supplementary file 1 [file pathogens-14-01286-s001.zip › Figure-s1-suplementary-material_pathogens-4030624.png]
